# Review of Zero-D and 1-D Models of Blood Flow in the Cardiovascular System

**DOI:** 10.1186/1475-925X-10-33

**Published:** 2011-04-26

**Authors:** Yubing Shi, Patricia Lawford, Rodney Hose

**Affiliations:** 1 Medical Physics Group, Department of Cardiovascular Science, Faculty of Medicine, Dentistry and Health, University of Sheffield, Sheffield S10 2RX, UK

## Abstract

**Background:**

Zero-dimensional (lumped parameter) and one dimensional models, based on simplified representations of the components of the cardiovascular system, can contribute strongly to our understanding of circulatory physiology. Zero-D models provide a concise way to evaluate the haemodynamic interactions among the cardiovascular organs, whilst one-D (distributed parameter) models add the facility to represent efficiently the effects of pulse wave transmission in the arterial network at greatly reduced computational expense compared to higher dimensional computational fluid dynamics studies. There is extensive literature on both types of models.

**Method and Results:**

The purpose of this review article is to summarise published 0D and 1D models of the cardiovascular system, to explore their limitations and range of application, and to provide an indication of the physiological phenomena that can be included in these representations. The review on 0D models collects together in one place a description of the range of models that have been used to describe the various characteristics of cardiovascular response, together with the factors that influence it. Such models generally feature the major components of the system, such as the heart, the heart valves and the vasculature. The models are categorised in terms of the features of the system that they are able to represent, their complexity and range of application: representations of effects including pressure-dependent vessel properties, interaction between the heart chambers, neuro-regulation and auto-regulation are explored. The examination on 1D models covers various methods for the assembly, discretisation and solution of the governing equations, in conjunction with a report of the definition and treatment of boundary conditions. Increasingly, 0D and 1D models are used in multi-scale models, in which their primary role is to provide boundary conditions for sophisticate, and often patient-specific, 2D and 3D models, and this application is also addressed. As an example of 0D cardiovascular modelling, a small selection of simple models have been represented in the CellML mark-up language and uploaded to the CellML model repository http://models.cellml.org/. They are freely available to the research and education communities.

**Conclusion:**

Each published cardiovascular model has merit for particular applications. This review categorises 0D and 1D models, highlights their advantages and disadvantages, and thus provides guidance on the selection of models to assist various cardiovascular modelling studies. It also identifies directions for further development, as well as current challenges in the wider use of these models including service to represent boundary conditions for local 3D models and translation to clinical application.

## Introduction

The cardiovascular system is the vehicle for the transport of blood throughout the body, conveying nutrients to the body tissues and organs and removing some waste products [1]. The circulatory system comprises the four chamber heart (including the four heart valves), the systemic vessels that deliver the blood to and collect the blood from the peripheral organs, and the pulmonary vessels that transport the blood through the lung for exchange of oxygen and carbon dioxide. The heart contracts to pump the blood into the systemic and pulmonary vasculature for circulation around the whole body, and the four heart valves maintain the direction of the flow. The systemic and pulmonary vessels each can be divided into aorta/pulmonary artery, main and small arteries, arterioles, capillaries, venules, veins, and vena cava/pulmonary vein. From the aorta to the arteries, arterioles, and on into the capillaries, the vessel branches into a tree-like structure, with vessel diameters decreasing, overall vessel luminal area increasing and the vessel wall becoming stiffer for every later generation of branching. The aorta and the larger arteries are quite elastic, and they act as reservoirs that buffer the pulsatility of the flow from the heart. Due to the changing vessel properties, as well as to bifurcations, there are pulse wave reflections in the system, which can have advantageous or disadvantageous effect on the heart loading and on coronary perfusion depending on the timing of the reflected wave in relation to the incident wave. There is also active control, in that the arterioles changed their vessel calibre under neuro-regulation and various bio-chemical regulations, to adjust the pressure and flow of blood to the peripheral organs. Capillaries are where the exchange of nutrients and metabolites take place. From the capillaries to venules, veins and vena cava, the vessels are merging along the flow direction to form an inverse tree-like structure, with greater compliance in the larger vessels. The vena cava and the larger veins are the most elastic vessels and they serve as a reservoir to accommodate the redistribution of blood volume during transitions between different physiological conditions. Some veins such as those in the lower extremity have venous valves to aid the directionality of the blood flow, and they are also subjected to the compression action of the surrounding muscles, providing a secondary pumping action returning blood to the right side of the heart. Some other veins such as those in the lung, collapse when their intra-luminal pressure is lower than the extra-luminal tissue pressure. These effects need special treatment during cardiovascular modelling. Generally the mean intra-luminal pressure and the pressure pulsatility decrease along the blood flow direction from arteries to veins, although in the aorta and larger arteries pulsatility can actually increase due to pulse wave reflections [2]. Detailed circulatory physiology is introduced in various physiology textbooks such as [1].

Like all fluid systems, blood flow in the cardiovascular system obeys the laws of mass conservation, momentum conservation and energy conservation, which are described by a group of governing equations. Unlike more traditional engineering piping networks the vessels are comparatively flexible, and the constitutive equations of the vessel walls provide additional constraints that strongly influence the blood flow dynamics. Furthermore the mechanical propulsion is provided by the muscle of the heart, governed by its own constitutive equations including active components.

A first division of the literature on cardiovascular dynamics research is the classification into time domain or frequency domain studies. Frequency domain representations of the cardiovascular system [3-7] are based on linearisation of the governing equations, achieved by neglecting the convective acceleration terms. The simplified equations are then solved in the frequency domain using Fourier or Laplace transformation. Frequency domain analysis permits fast and effective solution methods, but due to the linearization process it is more suitable for analysing problems in which the system is only slightly perturbated from a reference state. For a broader range of problems in which the nonlinear terms cannot be neglected, time domain methods must be employed. The focus of this review is on models represented and solved in the time domain.

**Figure 1:**
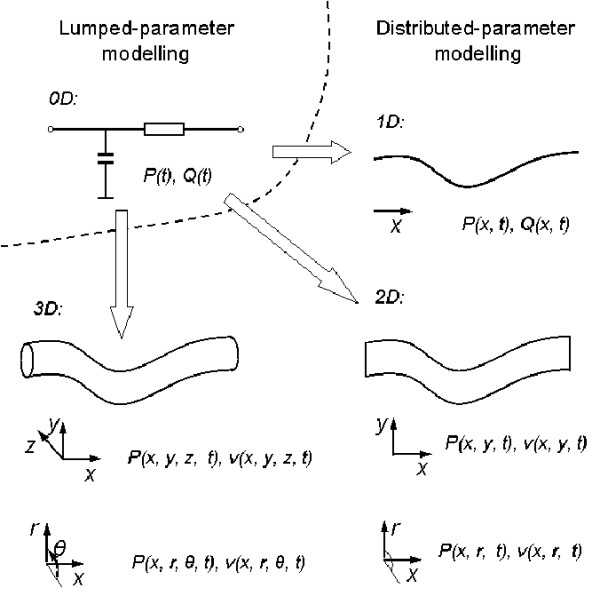
**Different scales of modelling**.

The selection of the appropriate dimensionality in a model representation, from 0D though to 3D, depends on the aims, and on the required accuracy, of the research study. Zero dimensional, or lumped parameter, models assume a uniform distribution of the fundamental variables (pressure, flow and volume) within any particular compartment (organ, vessel or part of vessel) of the model at any instant in time, whilst the higher dimensional models recognise the variation of these parameters in space. This is illustrated in Figure 1.

Zero dimensional models give rise to a set of simultaneous ordinary differential equations (ODEs): in representations of the vasculature there are often two ODEs for each compartment, representing conservation of mass and conservation of momentum, complemented by an algebraic equilibrium equation relating compartment volume to pressure. System models constructed from 0D components generally feature the major components of the system, such as the heart, the heart valves and compartments of the vasculature, and are suitable for examination of global distributions of pressure, flow and blood volume over a range of physiological conditions, including study of interactions between the modelled components. One, two and three dimensional models give rise to a series of partial differential equations describing conservation of mass and momentum (the Navier-Stokes equations), again complemented by equilibrium equations. In the context of cardiovascular mechanics, 1D models have the important facility to represent wave transmission effects within the vasculature: these are important in the aorta and larger systemic arteries. 3D solutions are required to compute complex flow patterns, for example in the ventricles, around heart valves, near bifurcations, or in any region with vortical or separated flows. No analytical solutions are available for any but the simplest geometries, and recourse is always made to numerical solution. In the context of the vasculature, there is a place for 2D models which can represent the radial variation of velocity in an axisymmetric tube. In 1D models, velocity is integrated over the cross-sectional area of the vessel to give flow based on some simplifying assumption about the velocity profile. Table 1 summarises the dimensionality of the fluid mechanics representations and their broad ranges of applications in the study of cardiovascular dynamics. 3D and 2D models are not the subject of the current review. For the state of the art in the 3D cardiovascular flow modelling, interested readers are referred to the review papers by [8] and [9].

**Table 1:** Comparison of modelling techniques for cardiovascular dynamics studies

Method of study			Suitable research target
Time	0D (lumped parameter) model		Global cardiovascular dynamics in the whole circulation system; General pressure and flow-rate changes in a local circulation loop; possibly to provide boundary conditions for local 3D models
	
domain	Distributed	1D	Pulse wave transmission; improved boundary conditions for 3D local models, capable of capturing systemic wave reflection effects
		
study	parameter	2D	Local flow field study in axisymmetric domains; further improvement of boundary conditions for local 3D models, but limited applicability
		
	model	3D	Local flow field study in full 3D domains

Frequency domain study			Frequency response analysis of cardiovascular system after linearization

It should be noted that when the 1D form of the Navier-Stokes equation is discretised in space and the convective term neglected, it becomes a series of 0D models.

## 0D cardiovascular models

0D models are developed to simulate the global haemodynamics in the whole circulation system. In carrying out 0D modelling, the concept of a hydraulic-electrical analogue is often applied. Generally, blood flow in the circulatory system and electric conduction in a circuit have much similarity: blood pressure gradient in the circulatory loop drives the blood to flow against the hydraulic impedance; similarly, voltage gradient in a circuit drives current to flow against the electric impedance. Hydraulic impedance represents the combined effect of the frictional loss, vessel wall elasticity and blood inertia in the blood flow, whilst electric impedance represents the combination of the resistance, capacitance and inductance in the circuit. Blood flow is described by the continuity equation for mass conservation, Poiseuille's Law for the steady state momentum equilibrium, and the Navier-Stokes equation for the unsteady state momentum balance; similarly the electric flow in the circuit is governed by the Kirchhoff's current law for current balance, and Ohm's law for the steady state voltage-current relation, and the transmission line equation for the high frequency voltage-current relation. Thus by representing the blood pressure and flow-rate with voltage and current, describing the effects of friction and inertia in blood flow and of vessel elasticity with resistance R, inductance L and capacitance C in the electric circuit respectively, the well-established methods for analysis of electric circuits can be borrowed and applied to the investigation of cardiovascular dynamics. The electrical analogue is unable to describe the nonlinearities that sometimes feature in cardiovascular mechanics including, for example, the contribution of the convective acceleration terms in the momentum equation and/or the nonlinear relationship between pressure and volume in a real vessel. Nevertheless these differences will not change the general nature of analysis when using the hydraulic-electric similarity, and when necessary these non-linearities can be specifically addressed in solving the governing equations.

0D cardiovascular system analysis started with the modelling of arterial flow using the famous Windkessel model. This was subsequently expanded to cover the modelling of other organs such as the heart, heart valves, and veins. The numerous 0D models that have been derived and reported to describe the specific characteristics of each of the circulatory subsystems are reviewed in this section.

### Models of the systemic vasculature

The first group of models considered is that which has been reported to describe the systemic vasculature, and for which the left ventricle and right atrium of the heart represents boundary conditions. Historically these models have evolved and increased in complexity as researchers have added components to capture particular physical or physiological phenomena. As stated previously, both the mean intra-luminal pressure and the pressure pulsatility generally decrease along the blood flow direction from arteries to veins (note that local increases of pulse pressure in the aorta and larger arteries due to wave reflection can be analysed using 1D models, but are not usually revealed in coarser 0D representations). There is a wide range of 0D systemic vasculature models to simulate the intra-luminal pressure and flow changes, from the simplest Windkessel model which considers the vein as a zero pressure sink and models the vasculature from aorta to capillary as a single capacitance C connected in parallel with a single resistance R, to the most comprehensive Guyton model [10] in which most of the main circulatory branches from arteries to veins were specifically represented as well as some autonomic and hormone regulation effects. These models can be divided into two subgroups, namely single- or mono-compartment models, in which increasing levels of sophistication are used to capture systemic response, and multi-compartment models, in which separate parts of the vasculature are represented as different compartments (often with similar electrical components but with different particular values of frictional loss coefficient, inertia and vessel compliance). Multi-compartment models even have the facility to capture wave reflections when the division of vessel segments is sufficiently small.

#### Mono-compartment descriptions

**Figure 2:**
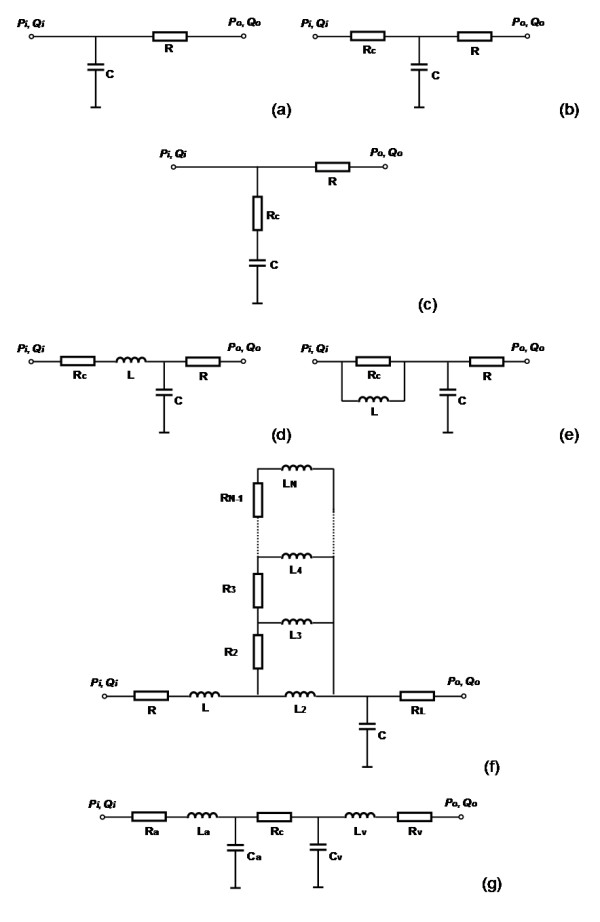
**Mono-compartment models for the vessel network**. (a) RC Windkessel model; (b) RCR Westkessel model; (c) RCR2 model; (d) RLCR1 model; (e) RLCR2 model; (f) RLCR1 model with sleeve effect; (g) RLCRCLR model.

In a mono-compartment description, the whole vessel network is described with a single resistance-compliance-inductance (RLC) combination (although there might be more than one of each component). The simplest and the first mono-compartment description is the famous two-element Windkessel model, which was first proposed by Stephen Hales in 1733, and later formulated mathematically by Otto Frank in 1899 [11]. The Windkessel model consists of two parallel elements, a capacitor C that describes the storage properties of large arteries and a resistor R that describes the dissipative nature of small peripheral vessels including arterioles and capillaries, as illustrated in Figure 2(a). This model was developed to represent the elementary characteristics of the systemic artery network, while the veins were neglected and represented as a far field zero pressure. Although it appears to be very crude compared with the more sophisticated models developed later, the two-element Windkessel model provides a simple way of representing the pressure decay in the aorta over the period of diastole. Even today, this simple RC combination model is still used in clinical practice for the estimation of total arterial compliance when peripheral resistance and the aortic pressure pulse waveform are known [1,11]. Despite the obvious restriction that this model has only a single time constant, and therefore cannot capture the high frequency components associated with pressure reflections in the artery network, it is nevertheless often used in cardiovascular modelling to provide a simple representation of the after-load on the heart. It is also often used to provide a simple terminal boundary condition in more elaborate distributed parameter representations.

Extending the Windkessel model for the simulation of arterial characteristics, Landes [12] introduced an extra resistance element R_c_, connected in series with the RC Windkessel model as shown in Figure 2(b). This model has been extensively studied by Westerhof and co-workers [13], and is sometimes called the Westkessel model, or RCR model. The second resistance R_c _represents the characteristic impedance of the arterial network, defined as the ratio of the oscillatory pressure and the oscillatory flow-rate when no reflective waves are present [2]. The overall resistance R_c_+R equals the total systemic vascular resistance in the previous RC model, and the capacitance C represents the elasticity effect of the arterial network [13]. Despite its simplicity, the introduction of the proximal R_c _greatly improves the high frequency performance of the model [13]. *In vivo *and numerical studies have indicated that the RCR model provides, subjectively, a good representation of after-load in the context of prediction of stroke volume, stroke work, and systolic and diastolic aortic pressure [14]. It is widely used in cardiovascular simulations as the after-load for the evaluation of cardiac function under various physiological and pathological conditions. However, *in vivo *studies have also indicated that the RCR model significantly underestimates peak aortic flow, slightly underestimates mean arterial pressure, and does not provide realistic aortic pressure and flow waveforms, when compared to a realistic arterial impedance model under the same simulated ventricular action [14].

In parallel to the Westkessel RCR model, Burattini and Natalucci [3] developed a different configuration of the three element RCR model to describe the arterial characteristics, in which the a small resistance R_c _was placed in series with the capacitor C instead of in series with the RC combination, as shown in Figure 2(c). In this configuration the small resistance R_c _was conceptually coupled with the capacitor C to describe the visco-elastic property of the vessel wall, in contrast to its use to capture grossly the wave reflection response in Westerhof et al.'s RCR model. By tuning the model parameters, the two RCR models could produce the same frequency characteristics and thus they were equivalent to some degree. However, it remains a problem of whether the visco-elastic property of the vessel has stronger influence on the arterial characteristics than the wave reflection and thus needs to be considered with priority.

Landes [12] further extended the RCR arterial model by incorporating the inertial effect of blood flow, forming a model configuration of RLCR1 as shown in Figure 2(d). Westerhof et al. also added the inertial effect of blood flow in the RCR model and proposed another four-element arterial model of RLCR2 [15,16], as illustrated in Figure 2(e). Inclusion of the inertial term L helps to further improve the modelling accuracy of vessel impedance in the middle frequency range. Several *in vivo *studies have been carried out to compare the modelling accuracy of the RC, RCR, RLRC1 and RLRC2 models, and it has been demonstrated that the RLRC1 model best reproduces the character of the vascular impedance data [17,18]. However, with more elements included, identification of model parameters becomes a difficulty. The model parameters are often obtained through nonlinear regression analysis based on measured pressure/flow data at aortic root. With only one more element added, parameter identification for the four-element RLRCR1 and RLRCR2 models are much more difficult than for the three-element RCR model and the two-element RC model. For this reason the RLRCR1 and RLRCR2 model are not so widely used as the RCR and RC models.

To further improve the arterial modelling, Westerhof et al. have also extended the RCR model by including more R and L components, configured as illustrated in Figure 2(f), to simulate the laminar oscillatory flow impedance, the so called sleeve effect [15]. This model is excellent for test cases representing Womersley's solutions for axisymmetric flow in a straight cylindrical tube, but has less relevance to complex vascular networks. It is relatively complicated, and accurate resolution of the radial distribution of velocity/flow can be better achieved through two dimensional or three dimensional computational fluid dynamics studies. As a result this model has been relatively unexploited by other researchers in the field.

The above RC, RCR and RLRC1 models were mainly developed to describe the pressure and flow characteristics in the aorta, and they were also applicable to arterial pressure/flow modelling in general vessel branches where the pressure and pressure pulsation in the venous side of the vessel beds are negligible, and this is the case for most of the applications. However, for some circulation beds such as coronary and pulmonary circulations, there are significant pulsatile components in the venous pressure and flow and thus the venous side contribution to the overall haemodynamics is not negligible [19,20]. To rectify the problem, more complex 5, 6 and 7 element vascular models (RCRCR, RCRCLR and RLCRCLR models) have been derived in which extra R, C and L elements were included to account for the dynamic characteristics of the veins. For the calculation, measured venous pressure or flow was applied at the venous side, in addition to the pressure or flow input at the arterial side, for the evaluation of overall response in the studied vessel branch [19,20]. Figure 2(g) shows a 7 element RLCRCLR model. Frasch et al. [20] carried out *in vivo *measurements and numerical data fitting, and demonstrated that, compared with the 2, 3, and 4 element Windkessel models, the 5 element RCRCR model provides a better description of microcirculation dynamics, and the 6 element RCRCLR model gives better representation of the dynamic contribution of the venous subsystem of the systemic vasculature. The specific characteristics of the 7 element RLCRCLR model in modelling the whole systemic circulation was not reported, but it was designed to further improve the mono-compartment description of the systemic vasculature network by improving the representation of the venous subsystem. Originally the 7 element RLCRCLR model was proposed as a mono-compartment model to describe the characteristics of the complete systemic vasculature, but we can also interpret it as a series connection of an RLC model for the arterial subsystem, a resistance model for the capillaries, and another RLC model for the venous subsystem. In this sense it can also be considered as a multi-compartment model as discussed in the next section.

#### Multi-compartment descriptions

In the mono-compartment models discussed above, the whole systemic vasculature is treated as a single block, and thus the internal distribution of pressure and flow-rate in the different segments of the vessel network is not computed (although, depending on the problem formulation, some spatial information might be computed as internal compartment variables). Multi-compartment models have been developed to address these shortcomings. In these models the systemic vasculature is partitioned into a number of segments, and each segment, or compartment, is described by its own resistance R, compliance C and inductance L, depending on the local vessel characteristics. The vessel segments are connected together to form the complete model of the whole vessel network. Depending on the specific aims of any particular study, and the requirement for modelling accuracy, the systemic vasculature can be appropriately partitioned to provide detail in the region(s) of interest, whilst other segments can reasonably be lumped together using less sophisticated model elements. This flexible and simple description of the systemic vasculature is a powerful tool for cardiovascular simulation.

In constructing the multiple compartment models for the vessel network, it is necessary to first derive suitable RLC models for the vessel segment, as a building block in the development of the whole vessel network model. It may be argued that the mono-compartment descriptions such as RC, RCR and RLCR models discussed in the previous section can be directly used for this purpose, and indeed some researchers have adopted these descriptions [21-24]. However these configurations have been developed to provide appropriate descriptions for the systemic vasculature as a unit, and are not necessarily the most appropriate for its component parts. The characteristics of the vessel segments are in general quite different from those of the system. Formaggia and Veneziani [25] and Milisic and Quarteroni [26], after reviewing existing descriptions, provided detailed derivations of four typical compartment model configurations appropriate for the description of a vessel segment. The derivation considered the blood flow in a vessel segment as a one dimensional formulation, and mean flow-rate and pressure over the whole vessel segment were assumed to be equivalent to either the input or the output value (the choice for each depending on the specific formulation). The four compartmental configurations were labelled as L network element, inverted L network element, T element and π element as illustrated in Table 2. Each is most appropriately used with particular combinations of boundary conditions. Among these configurations, the inverted L network element uses the inlet flow and the outlet pressure as boundary conditions, and solves for the inlet pressure and the outlet flow. This is consistent with the common practice of using upstream velocity and downstream pressure as boundary conditions in 2D and 3D computational fluid dynamics studies.

**Table 2:** Four typical vessel segment models as building blocks of multi-compartment description of the vessel network (after [25,26])

Network element	Circuit model	Corresponding boundary conditions
Inverted L element		Upstream flow-rate *Q*_*i *_and downstream pressure *P*_*o*_

L element		Upstream pressure *P*_*i *_and downstream flow-rate

T element		Upstream and downstream pressures *P*_*i *_and *P*_*o*_

π element		Upstream and downstream flow-rates *Q*_*i *_and *Q*_*o*_

**Figure 3:**
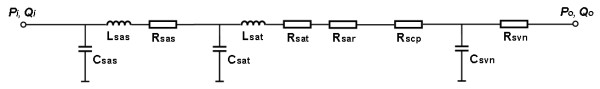
**A sample multi-compartment model**. (sas: aortic root; sat: artery; sar: arteriole; scp: capillary; svn: vein).

Using these network elements as building blocks, several multi-compartment models of the systemic vasculature have been developed, with various levels of complexity, from the single branch models upwards. Most researchers take the approach of partitioning the systemic vasculature into segments representing aorta, artery, arteriole, capillary, and vein [27-32], characterising the network element to suit local flow features, and then connecting the segments to form the circulation loop. In the aorta and the main arteries the blood vessels are quite elastic, and the blood flow is pulsatile, thus the full resistance, compliance and inductance effects (RLC combination) need to be considered. In the arterioles and capillaries, the vessel wall is relatively rigid, the flow is steady and frictional loss is the dominant factor, thus the local flow dynamics is adequately described by a pure resistance element. The general veins and vena cava are compliant and the blood flow is relative steady, thus the inertial effect is often neglected and an RC combination is considered sufficient to describe their flow characteristics. It is understood that some peripheral veins, such as those in the lower extremities, are under the compression action of the surrounding muscles as well as having valves to keep the one-way flow direction. However, in studying the dynamics of the whole circulatory system, such local scale effects are secondary when compared with the more dominant contribution of the vena cava and pulmonary vein as blood reservoirs, thus the RC combination provides an appropriate characterisation for general veno-dynamics. Figure 3 illustrates a sample multiple compartment model for the systemic vasculature developed by Shi et al. [33]. Some researchers have also built multi-branched multi-compartment models [22,23,34-44] to investigate blood flow distribution and pressure/flow characteristics in each simulated vessel branches. It is the opinion of the authors of this review that there is a danger in the adoption of these more complex descriptions that, although in principle they can represent the system more accurately, in practice it is often very difficult to estimate appropriate values for many of the model parameters. Estimation of more parameters requires more measurements, and solution of the inverse problem to estimate the parameters even if measurements are available is often non-trivial. Noordergraaf et al. [43], Avolio [45], and O'Rourke and Avolio [44] have constructed full, frequently cited, models for the systemic arterial network. O'Rourke and Avolio's model bore a close relationship to Noordergraaf et al.'s model, but was claimed to be more realistic in its representation of the vascular bed, particularly that in the upper part of the body [44].

#### Summary of properties and attributes of systemic vasculature models

As a summary, the vessel models described above are compared in Table 3.

**Table 3:** Comparison of various 0D models for the systemic vasculature

Model configuration		Advantages	Disadvantages		
	RC model	Reveals the general storage properties of large arteries and the dissipative nature of small peripheral vessels with the simplest model structure	Cannot simulate the effect of high frequency components in the arterial impedance, can not accurately match the aortic pressure and flow-rate waveforms	Venous pressure is assumed to be zero and thus venous pressure fluctuations cannot be described.	Cannot describe the pressure and flow-rate changes in specific segments of the vasculature; cannot simulate the pulse wave transmission effect
			
Mono-compartment model	RCR model	Simple, and gives a better description of the high frequency components in the arterial impedance than the RC model	Can not describe the features of the secondary maximum and a discrete minimum in the medium frequency range of the arterial impedance.		
			
	RLCR model	Simple, and offers improved description of the secondary maximum and a discrete minimum in the medium frequency range of the arterial impedance than the RCR model	Parameter setting is more difficult than for the RCR and RC models, which limited its applications.		
			
	RLCRCLR model	Simplest model that accounts for venous pressure fluctuations	The model structure is complex compared with RC, RCR and RLCR models, thus parameter setting is more difficult.		

Multiple compartment model		Flexible combination of RLC network elements to describe the vessel characteristics to whatever level of detail required. Captures, within the limitations of the model, pulse wave transmission effects.	More complex to implement than the mono-compartment models. Difficult to determine appropriate RLC parameters when the model includes many vessel segments.		

### Models of the heart

#### Isolated chamber models

There have been numerous studies on quantitative characterisation of the heart as a pump. Leefe and Gentle [46] discussed the characteristics of the left ventricle, exploring whether it was better described as a pressure or as a flow source: of course in truth it is a combination of the two. In the 1970s, Suga et al. [47] proposed a varying elastance model for the ventricle. In this model, the ventricular pressure is presented as a function of the ventricular elastance and the change of the ventricular volume from its unstressed value. The change of ventricular volume is determined by the blood flow into and out of the ventricular chamber, and the ventricular elastance is defined as a time-varying function based on the *in vivo *measurement of the ventricular activity over the cardiac cycle. This model is easy to understand and to implement, and it has been widely adopted by researchers such as [23,27-29,32,35,48-51].

Various alternatives to the varying elastance model have been described. Zacek and Krause [31] derived a heart model in which heart muscle mechanics were based on Hill's three parameter model. The ventricular pressure was calculated from the computed muscle force and the volume calculated from the change of muscle length. Werner et al. [42] proposed equations to calculate the myocardial wall tension in systole and diastole by using Hill's model and considering the Frank-Starling effect, and then calculated the ventricular pressure based on the Laplace law by assuming the heart chamber to be spherical in shape. Bovendeerd et al. [52] and Diaz-Zuccarini and LeFevre [53] calculated the ventricular pressure in a similar way. Another simpler model for the heart is to use an exponential equation to define the cardiac output as a function of the atrial pressure, with ventricular dynamics details completely neglected [54,55]. Nevertheless, due to its concise model structure and clear physical meaning, the varying elastance model remains the most popular. In addition to its original usage as a descriptor of left ventricle performance, the variable elastance model has also been extended for applicability to the simulation of atrial dynamics [29,32,35,38,50,51]. Yaku et al. extended the variable elastance model further to study ventricular fibrillation, assigning different parameters to the normal and diseased cardiac muscles [49].

#### Models of Chamber Interactions

The models described in the previous section treat the heart chambers in isolation. Various interactions including atrial-ventricular interaction [56,57], ventricular interaction [29,32,36,38,58-63], and the effect of the pericardium on the cardiac dynamics [29,36,38,59,61] have also been studied. Noting that the atrial-ventricular septum (annulus fibrosus) undergoes large displacements during the heart cycle, and that the septum motion contributes about 10% of the cardiac output, Korakianitis and Shi [56,57] have derived detailed equations to model the septum motion. Ventricular interaction was also considered to be important in cardiovascular dynamics studies by some researchers [29,32,36,38,58-63]. The left and right ventricles are separated by a ventricular septum. During diastole, the imbalance of pressure in the two ventricular chambers produces septum motion, thus causing an interaction in the filling of the two ventricles. In systole the septum also contracts and contributes to the cardiac output. To model this process the variable elastance model has been further developed to account for the specific response of the left and right ventricular free walls and the ventricular septum. Another interaction extension is to include the effects of the enclosure of the heart in pericardium, recognising that the volume change in one heart chamber would affect the pressure value in the pericardial sac, and thus that the pressure-volume relation in the other three chambers would inevitably be affected. To model this feature, an exponential pressure-volume relation was assumed in the sac, and applied in the calculation of pressure-volume relations in the four heart chambers [29,36,38,59,61].

These interactions were proposed and studied, but further validation was still lacking due to the complexity of the problem and limitations of experimental technique, thus there is little reference to them in the most recent literature.

### Models of the heart valves

There are four heart valves in the normal heart, the mitral, tricuspid, aortic and pulmonary valves. The valves prevent backflow of blood from the ventricles to the atria during systole (mitral and tricuspid valves) or from the aorta and pulmonary arteries into the ventricles during diastole (the aortic and pulmonary valves). These valves close and open passively under various external effects of pressure gradient across the valve, vortex flow near the valve [64,65], shear force acted on the valve leaflet surfaces etc. Extensive analytical, numerical and experimental studies [66-68] have been carried out to investigate valve dynamics in three dimensions, but the fundamental mechanics of opening and closure remain difficult to characterise.

The simplest models of the heart valve used in 0D studies of cardiovascular dynamics, featured the valve as a diode plus a linear or nonlinear resistance [22,50,69,70]. The valve has little resistance to the flow when the pressure gradient across it is positive, while the flow is totally stopped when the pressure gradients across it is negative. This idealised description ignores the more complex features of valve dynamics. The complexity of valve motion has been demonstrated *in vivo*: for example, Leyh et al. [71] conducted trans-thoracic and trans-oesophageal echocardiographic studies on 20 human subjects after different surgical interventions for repair of aortic valves, and found that the valve undergoes a three-stage motion pattern: a rapid early systolic opening, a slow middle systolic closing, and a rapid early diastolic closing movement. Clinical observation has also revealed that the mitral valve has a regression motion that causes the leaflet to return to the fully open position before the rapid early diastolic closing [1,72].

It is clear that real valve motion is a more complex procedure than a simple change of status between open and closed as described by the idealised diode model. Zacek and Krause [31] considered the change of heart valve resistance during valve motion by using the concept of a time-dependent drag coefficient. In their work the drag coefficient was a prescribed function of the valve open area, and it approached infinity when the valve was closed. The drag coefficients were added to the losses of the conduit in which the valve was situated. Werner et al. [42] described the valve behaviour by including the volume of the reverse flow during the closure phase: in their study this was referred to as the 'dead space volume', which was a function of the valve leaflet opening angle and became zero when the valve was fully closed. Shi et al. [73] modelled the valve dynamics by considering the local flow resistance and the blood inertial effect. The valve was described with an orifice model, and the valve opening change was prescribed based on previous experimental observations. Although each of these models has merit in representing further details of valve behaviour, in all cases this behaviour was prescribed whilst the underlying valve dynamics and motion mechanism were not considered. As a result, none could simulate the three stage motion pattern of the valve revealed by [71]. To further improve the valve dynamics modelling, Korakianitis and Shi [74] proposed a more advanced heart valve model, in which the valve dynamics were described by an ordinary differential equation that considered the different effects of pressure gradient across the valve, vortex flow near the valve, shear force acted on the valve leaflet surfaces etc. In this model, the relative importance of these factors was determined by referring to the results of previous two dimensional and three dimensional computational fluid dynamics studies. With these improvements, the model could effectively simulate the valve opening and closing procedures, and the numerical results agreed well with the published results on *in vivo *measurement of valve motions with echo-cardiography [1,71,72].

### Nonlinear effects and external interactions

Although the electrical-hydraulic analogue has been widely used to study cardiovascular dynamics it should be noted that, in contrast to the electric system, the cardiovascular system can exhibit strong nonlinearities. These nonlinear effects include sympathetic/parasympathetic neuro-regulation, auto-regulation in cerebral and coronary circulation loops, cardio-pulmonary interaction, collapse of vessels due to environmental pressure, effect of venous valves, and pressure-dependent vessel compliance in the artery etc.

#### Neuro-regulation

The nervous system has an important influence on the cardiovascular changes, through the sympathetic and para-sympathetic nerves. There are baroreceptors in the aortic arch, carotid artery and atrium, which are continuously monitoring blood pressure and blood oxygen levels. When these physiological variables are out of the normal ranges, the baroreceptors trigger the nervous system so that sympathetic and/or parasympathetic nerves are stimulated and regulate cardiovascular response. The sympathetic nerves can trigger arteriole contraction and thus cause an increase in resistance to blood flow and a concomitant decrease in the rate of blood flow through the tissues. Similarly it can decrease the unstressed volume of veins and thus push more blood into the heart for circulation: this in turn causes an increase in heart rate and an increased cardiac pressure and cardiac output. In contrast, action of the parasympathetic nerves causes a marked decrease in heart rate and a slight decrease in heart muscle contractility. Through these mechanisms the nervous system controls the circulation by redistributing blood flow to different areas of the body, increasing or decreasing pumping activity by the heart, and, especially, providing very rapid control of systemic arterial pressure [75]. Neuro-regulation can increase arterial pressure to double its normal value within 5 to 10 seconds and, conversely, sudden inhibition can decrease the arterial pressure by 50% within 10 to 40 seconds. Thus in some physiological conditions, such as posture change or tilting, hypoxia, response to gravitational acceleration, effect of intra-thoracic pressure variation etc., the effect of neuro-regulation on the cardiovascular system becomes too important to be neglected.

Ursino et al. have developed an analytical model of neuro-regulation, and combined this model with a multiple compartment cardiovascular model for the simulation of physiological and pathological responses under various conditions of isocapnic hypoxia [41,76], haemorrhage [40], hypercapnia and hypocapnic hypoxia [77] and carotid occlusion [27]. Lu et al. [35] derived detailed models for a fast vagal pathway and three slow sympathetic pathways for the control of heart rate, myocardial contractility and vasomotor tone, and combined the neuro-control model with a cardiovascular system model and a lung mechanics model for the simulation of the cardiopulmonary response under the Valsalva manoeuvre. Green and Miller [78], using an RCR model to describe the systemic vasculature, adopted a simple linear equation to model the neuro-regulation effect of systemic compliance based on arterial pressure, and simulated cardiovascular response to acceleration stress. Melchior et al. [79] produced a comprehensive review of the mathematical modelling of the human cardiovascular system for the simulation of orthostatic response, including neuro-regulation.

#### Auto-regulation

In any tissue of the body, an acute increase in arterial pressure causes an immediate rise in blood flow. Within less than a minute, however, the blood flow in most tissues returns almost to the normal level, even when the arterial pressure remains elevated. This restoration of flow towards the normal state is called auto-regulation. In contrast with neuro-regulation, which is under the control of the central nervous system, auto-regulation is a local biochemical procedure. The detailed underlying mechanism of auto-regulation is unknown, but the metabolic requirement of the organ and the myogenic response of the vascular smooth muscle are considered to be two of the main causes [75]. Auto-regulation has an important influence on the blood flow in several local circulation loops, including the cerebral, renal, and hepatic circulations, and is a requisite component of any model of these subsystems.

Lodi and Ursino [80] have published a circulation loop model for the simulation of cerebral circulation dynamics. Based on the metabolic requirement theory for the auto-regulation effect, a sigmoidal auto-regulation curve was developed to relate the pial artery compliance to the cerebral blood flow, and the pial artery resistance was regulated indirectly by the changes in its volume associated with its compliance. Similarly Jeays et al. [81] developed a model based on myogenic response for the modelling of gut blood flow regulation and postprandial hyperaemia. Cornelissen et al. [82,83] modelled the auto-regulation effect in coronary circulation by considering the myogenic, flow-dependent, and metabolic flow controls. However, because the underlying mechanisms governing the auto-regulation effect are still under investigation, these modelling efforts are mostly based on incomplete assumptions.

#### Interaction between the cardiovascular system and the respiratory system

The main organs of the cardiovascular system (heart, aorta, and vena cava) and those of the respiratory system are all situated in the thoracic cavity. For this reason strong interaction exists between the two systems. In modelling the interaction between the cardiovascular system and the respiratory system, Bai et al. [84] developed a combined model for the simulation of cardio-pulmonary response in step-leap respiration exercise for the treatment of cor pulmonale. To account for the respiratory effect on the pulmonary circulation, the volumes of the pulmonary artery and veins were modelled using an exponential function of the intra-thoracic pressure. Lu et al. [35] combined a neuro-control model, a cardiovascular system model and a lung mechanics model for the simulation of cardiopulmonary response under the Valsalva manoeuvre. This model included specific consideration of the effects of pleural pressure on the intra-cardiac pressure and the pressures within the large intra-thoracic blood vessels, and the effect of lung air volume change on the capillary resistance in the pulmonary blood vessels. Lazzari et al. [85] built a cardiovascular model to study the interaction of a ventricular assist device and artificial ventilation, in which the influence of intra-thoracic pressure on the blood flow in the heart, systemic thoracic veins, and pulmonary vessel network was adequately considered.

#### Venous collapse due to environmental pressure

Large veins usually have little resistance to blood flow when they are distended. However, due to the compression action of surrounding tissues and organs, and to the rest state of intra-abdominal and intra-thoracic pressures, veins are usually at least partially collapsed. In these situations, the large veins do usually offer some resistance to blood flow [75]. The majority of published cardiovascular studies have focused on the cardiac and arterial systems, whilst veno-dynamics has received less attention,

Fung [86] produced a detailed analysis of venous collapse, and derived the detailed analytical formulations that govern the behaviour of the veins in the collapse phase. In the context of lumped-parameter modelling of venous collapse, Lu et al. [35] used an exponential pressure-volume relation for the modelling of the capacitance of systemic veins, with nonlinear and/or piecewise linear equations to describe the compliance and the resistance of the vena cava as functions of luminal blood volume. Zervides et al. [87] derived pressure-dependent venous resistance, compliance and inductance. Beyar et al. [29] modelled the venous resistance as linear function of the difference between the peripheral and central vena cava pressures when the peripheral venous pressure is less than zero, and as a constant value when the peripheral venous pressure is greater than zero. Ursino et al. [27,28] modelled the pressure-volume relation of the peripheral and venous vessels as a nonlinear exponential function. In simulating venous circulation, Snyder and Rideout [37] applied a higher compliance value (by twenty times) for the collapsed veins compared with that for un-collapsed ones, determining the current state by comparison of the current volume of a venous segment with its unstressed volume. Peterson et al. [24] applied a similar model in the simulation of the influence of gravity and posture on cardiac performance. In simulating the interaction between the native cardiovascular system with an intra-aortic balloon pump, Barnea et al. [48] represented the pressure-volume relation of systemic veins by a fifth order polynomial approximation.

#### Effect of venous valves

The valves in the veins are arranged so that the direction of blood flow (except for local and temporary changes due to venous compliance) can be only towards the heart. Every time a person moves the legs or even tenses the leg muscles, a certain amount of venous blood is propelled toward the heart. Thus valves in the veins have an important role in countering the tendency of some changes in posture (and associated gravitational changes) to cause flow away from the heart [75]. It has already been stated that veno-dynamics has received relatively little attention from the physiological modelling community: venous valves have received even less. Zervides et al. [87] modelled the venous valve as a diode to allow no backflow, and also reported on the effects of simple modifications to account for swept-volume flow reversal and for a leaky valve. Beyar et al. [29] simulated the venous valve by assigning different resistance values to the valve subjected to positive or negative pressure gradients. Similarly, Snyder and Rideout [37] simulated the venous valve by a two diode configuration in which the resistance to the reverse flow was different from that for the forward flow. Further improvements is possible by adapting more advanced models, such as the one developed by Korakianitis and Shi [74] to represent heart valve dynamics, for the simulation of venous valves.

#### Pressure-dependent constitutive equations and vessel properties

Most often in the 0D model descriptions and solution methods, the component values (R, L and C) are taken to be constant. However, since these represent real physical parameters they are subject to the same nonlinearities as any other description of vascular mechanics, namely geometrical and material nonlinearities. As the vessel diameter changes under changes of pressure, its compliance will change as will its resistance to flow. These effects can be included in 1D models, but are usually neglected in 0D models. The vessel wall exhibits a nonlinear stress-strain curve [88], meaning that the compliance C is also a function of the luminal pressure. Furthermore the vessel wall material is visco-elastic. However, given that the diameter changes in the arterial system are relatively small (order of 10%), and that the range of arterial pressures over the cardiac cycle is such that the material tends to operate in a relatively linear region of the stress-strain curve, it is possibly justifiable to neglect the pressure dependence of the arterial properties. This is not so for the veins, at least when they enter a collapsed state.

To address the pressure-dependence of the constitutive equations for vessels, several researchers have proposed models based on *in vivo *measurements or on theoretical derivations. Ursino et al. [27,28] used a linear pressure-volume relationship to model the arteries in the simulation of carotid baro-regulation of pressure pulsation. Fogliardi et al. [89] carried out *in vivo *experimentation to further test the linear and nonlinear formulation of the RCR model, and based on the experimental results commented that no additional physiological information was gained when a pressure-dependent compliance throughout the heartbeat was incorporated in the three-element Windkessel, compared with that using a constant compliance. They also reported that the nonlinear model did not significantly improve the approximation of diastolic pressure in the presence of an evident oscillation. In contrast, Li et al. [90,91] adopted an exponential variation of arterial compliance with pressure changes, applied the proposed relation in a three element RCR vessel model, and came to the conclusion that a pressure-dependent compliance could more accurately predict aortic and stroke volume. Cappello et al. [92] developed a one-step computational procedure for estimating the parameters of the nonlinear RCR model of the arterial system incorporating a pressure-dependent compliance.

Coronary vessels are subject to a different mechanical environment compared with that of other arterial vessels, associated with the large myocardial stresses generated in systole. For the simulation of coronary vessels Geven et al. [93] modelled the resistance of the coronary capillary bed as a linear function of left ventricular pressure in systole, and as a constant value in diastole. Bovendeerd et al. [52] used 20 times larger values for the coronary arterial, coronary myocardial and coronary venous resistances in normoxia compared to those in hyperemia. Barnea et al. [48] modelled the coronary arterial resistance as a linear function based on the pressure difference between the aortic pressure and venous pressure and the pressure difference between the aortic and ventricular pressure. Smith et al. [94] modelled the resistance and the compliance of the coronary arterioles, capillary and venules as function of both arteriolar and venular pressures. Spaan et al. [95] developed a model for the coronary loop in which the resistances of the coronary arteriole and veins changed under auto-regulation, and the same group [82,83] added myogenic, flow-dependent, and metabolic flow control to coronary flow modelling. The developed model has been validated [96,97] through *in vivo *measurements.

In summary, it might be deemed necessary to consider the pressure-dependent vascular properties in the coronary vessels and in the veins, but the argument for their usage in other arteries is less compelling.

### Integrated cardiovascular system models

**Figure 4:**
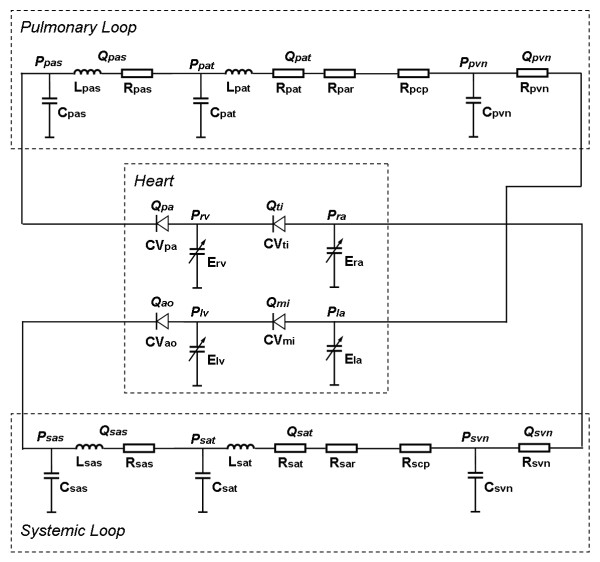
**A sample complete circulatory system model**. (sas: systemic aortic root; sat: systemic artery; sar: systemic arteriole; scp: systemic capillary; svn: systemic vein; pas: pulmonary artery root; pat: pulmonary artery; par: pulmonary arteriole; pcp: pulmonary capillary; pvn: pulmonary vein; lv: left ventricle; la: left atrium; rv: right ventricle; ra: right atrium; mi: mitral valve; ao: aortic valve; ti; tricuspid valve; pa: pulmonary valve).

Numerous 0D integrated system models have been developed by assembling component models of the vasculature, the heart and the heart valve, according to the needs of particular studies. The simplest system models represent the whole vasculature as two element (RC) or three element (RCR) Windkessels [53,69,78,91,98] whilst more comprehensive models feature multiple vascular compartments [27-32], sometimes with the important local branches represented individually [22,23,34-42]. The most common model of the heart in an integrated system model is the varying elastance model and the most common heart valve model is a simple diode. Figure 4 shows a typical integrated cardiovascular system model developed by Shi and Korakianitis [99].

A special case of 0D modelling is that of the specific study of the local circulation characteristics in some important vascular subsystem, such as cerebral, coronary, renal or lower extremity, where often multiple compartment models have been designed to include such features as complex anastomoses, auto-regulation effects and sometimes collapsible vessels and internal valves. Most commonly these detailed local simulations have not included a heart model, and usually flow rates and/or pressures are directly applied as boundary conditions. Examples of these types of models are those proposed by [23,37,52,80,93,100-104].

### Parameter settings of 0D models

Theoretically the model parameter in the 0D models can be derived from the pressure/flow data measured in selected positions of the circulatory system. However, several difficulties make parameter setting a challenging task. The invasive nature of many of the measurements, restricted access to the required measurement sites due to anatomical configuration, practical difficulties in the orientation of flow probes (particularly invasive ones), sometimes difficulties in synchronisation of pressure and flow data (particularly when not measured simultaneously), limited precision in the pressure/flow sensor, all contribute to the accuracy of the model parameters obtained. Perhaps more importantly, the pressure/flow measurement data available provide only part of the information needed for the estimation of a number of model parameters. Since a 0D model is an abstraction of the vasculature and it is not an exact mapping of the circulatory anatomy, it is hard to locate other anatomical positions in the vasculature in order to carry out extra pressure/flow measurement and provide further useful information for the refinement of the model structure and parameters. Thus with insufficient pressure and flow data as input, more than one set of values for the model parameters can satisfy the input condition, and it is hard to tell which set of parameter values is the right one for the model developed. Furthermore, derivation of model parameters depends on combined frequency domain processing and time domain multi-variable linear/nonlinear regression analysis. When the model structure is simple and the number of model elements (i.e., the number of R, L and C components) is small, the regression analysis is simple and can be linearized sometimes. As the model becomes more sophisticated with more elements included, usually the regression analysis cannot be linearized. In this situation finding a proper parameter combination for the model is quite difficult, and the regression analysis often produces poor results even if converged results can be obtained. The only solution for such a situation is first to find the reasonable ranges of each parameters, and then assign different value combinations to the model parameters from the identified reasonable ranges and observe whether the produced response match the measured pressure/flow data. This trial and error procedure is repeated until an acceptable value combination of parameters is obtained. This procedure is very time consuming, and the results obtained are only correct in the sense of mathematical matching, with no guarantee of a robust physical interpretation.

Different implementations of this procedure have been extensively designed and applied in the identification of 0D cardiovascular model parameters [4,18,89,90,92,105-111]. The obtained model parameters and the experimental measurement data were adapted for parameter setting in the 0D simulation models. Depending on the factors including the difference in model structure and model complexity, different healthy/diseased conditions studied, accuracy of in vivo measurements as input for model identification etc., the model parameters adopted by different researchers showed enormous difference. As an example of the parameter ranges, Table 4 shows the parameter values suggested by some researchers for the simple RC, RCR and RLRC arterial models. For comparison Table 5 illustrates some experimental data for similar model configurations. The huge variation among the model parameters shown in these tables represents the inter-group modelling error among researchers, as well as the inter-patient differences. For more complex vascular models and cardiac models their parameter settings show even wider scattering (interested readers may refer to the literature list in the above sections for details). It is necessary to develop more accurate and efficient techniques to optimise the parameter setting in 0D models.

**Table 4:** Some published model data adopted by previous researchers in their human systemic arterial models

Source	Model	Model parameters
Cavalcanti and Belardinelli [98]	RCR	Original values: *R*_c _= 52 *dyn *· *s*/*cm*^5^, *R *= 1200 *dyn *· *s*/*cm*^5^, *C *= 0.001 *cm*^5^/*dyn* Converted into physiological units: *R*_c _= 0.039 *mmHg s*/*ml*, *R *= 0.09 *mmHg s*/*ml*, *C *= 1.333 *ml*/*mmHg*

Cole et al. [165]	RC	*R *= 0.7 *mmHg s*/*ml*, *C *= 3.1 *ml*/*mmHg*,

Cole et al. [165]	RCR	*R*_c _= 0.03 *mmHg s*/*ml*, *R *= 0.7 *mmHg s*/*ml*, *C *= 3.1 *ml*/*mmHg*

Cole et al. [165]	RLCR1	*R*_c _= 0.028 *mmHg s*/*ml*, *R *= 0.65 *mmHg s*/*ml*, *C *= 2.8 *ml*/*mmHg*, *L *= 0.0018 *mmHg/(ml s*)

Cole et al. [165]	RLCR2	*R*_c _= 0.045 *mmHg s*/*ml*, *R *= 0.63 *mmHg s*/*ml*, *C *= 2.53 *ml*/*mmHg*, *L *= 0.0054 *mmHg/(ml s*)

Lerma et al. [166]	RCR	Original values: *R*_c _= 57 *dyn *· *s*/*cm*^5^, *R *= 1332 *dyn *· *s*/*cm*^5^, *C *= 0.0007 *cm*^5^/*dyn* Converted into physiological units: *R*_c _= 0.043 *mmHg s*/*ml*, *R *= 1 *mmHg s*/*ml*, *C *= 0.9332 *ml*/*mmHg*

Segers et al. [167]	RCR	*R*_c _= 0.124 *mmHg s*/*ml*, *R *= 1.28 *mmHg s*/*ml*, *C *= 1.01 *ml*/*mmHg*

**Table 5:** Some published experimental data from previous researchers for human systemic arterial vasculature models

Source	Model	Values in physiological units
Liu et al. [106]	RC	*R = *0.89 ~ 1.99 *mmHg*· *s*/*ml, C = *0.47 ~ 1.86 *ml/mmHg,*

Segers et al. [168]	RLCR2	*R*_*c *_*= *0.035 ~ 0.109 *mmHg*· *s*/*ml, R = *0.82 ~ 1.15 *mmHg*· *s*/*ml, C = *0.68 ~ 1.32 *ml/mmHg, L = *0.005 *mmHg/(ml*·*s)*

Stergiopulos et al. [16]	RCR	In vivo measured: *R*_*c *_*= *0.058 ~ 0.070 *mmHg*· *s*/*ml, R = *0.63 ~ 0.79 *mmHg*· *s*/*ml, C = *1.28 ~ 2.48 *ml/mmHg* Data fitted: *R*_*c *_*= *0.03 ~ 0.033 *mmHg*· *s*/*ml, R = *0.63 ~ 0.79 *mmHg*· *s*/*ml, C = *1.75 ~ 5.16 *ml/mmHg*

Westerhof et al. [13]	RCR	Original values: *R*_*c *_*= *90 *g*/(*cm*^4 ^*s*), *R = *1200 *g*/(*cm*^4 ^*s*), *C *= 8 × 10^-4 ^*cm*^*4 *^*s*^2^/*g* Converted into physiological units: *R*_*c *_*= *0.0675 *mmHg*· *s*/*ml, R = *0.9 *mmHg*· *s*/*ml, C = *1.067 *ml/mmHg*

### Applications of 0D models

**Figure 5:**
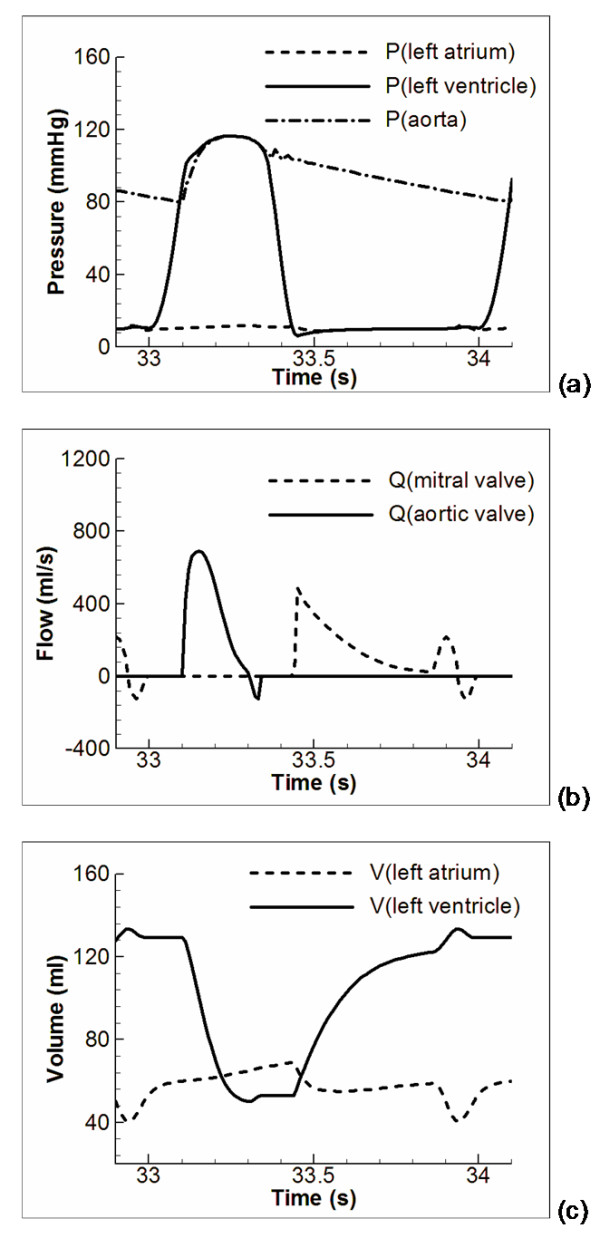
**Sample cardiovascular response in the systemic loop in a healthy human subject simulated with 0D models**. (a) Pressure changes; (b) Flow changes in the two heart valves; (c) Volume changes in the two left heart chambers.

Research on the 0D modelling of the cardiovascular system has continued for more than two hundred years since the proposition of the Windkessel concept. 0D models are widely used in various areas of cardiovascular studies, from basic cardiovascular physiology research to astronautic medicine and design analysis of cardiovascular artificial organs. To illustrate application of a 0D model, Figure 5(a) to 5(c) presents some exemplar 0D model simulation results, including the pressure, flow and volume changes in the systemic circulation of a healthy human subject. The full model details and the parameter settings can be found in [112]. These simulation results agree well with those published cardiovascular response such as in [72,75,113,114]. Typical applications of 0D cardiovascular models have been extensively referred to in the previous section. For comparison they are summarised in Table 6.

**Table 6:** Typical applications of 0D model

Application	Model feature	Examples
Analysis of the systemic arterial flow characteristics	Only the arterial network is modelled	Characteristics of the three- and four-element Windkessel models [13,15,16,43,114,167,169,170]; Advantages and disadvantages of using the three element RCR model as aortic input impedance [14]; Comparison of different configurations of three element and four element models as the embryonic aortic impedance [110]; Investigation of aortic input impedance in infants and children by curve fitting to two, three and four element Windkessel models [17]; Study of the linear and nonlinear formulations of the three element Windkessel model by considering the pressure-dependent capacitance effect in the arterial network [89,90,92]; Two port analysis to extend the Windkessel models by considering the venous side flow pulsations in the systemic loop [19,20]; Ventricular-systemic arterial coupling [171].

Hemodynamic response in the native cardiovascular system under various healthy and diseased conditions	Complete description of the native cardiovascular system	Cardiovascular response in normal healthy subjects [31]; Study of the ventricular interaction effect [32]. Modelling the dysfunction in regional stunned myocardium of the left ventricular [69]; Modelling of cardiac muscles in the study of mechanics and energetics of fibrillating ventricle [49]; Study of changes in pulmonary venous pressure after the onset of left ventricular dysfunction [30].

Hemodynamic changes under various surgical and therapeutical interventions.	The native cardiovascular system was partly changed.	Circulation dynamics in the presence of the bidirectional cavopulmonary anastomosis in children with a uni-ventricular heart [50]; Rest and exercise hemodynamics in patients with total cavopulmonary connection [172]; Modelling the hemodynamic characteristics in patients with hypoplastic left heart syndrome after the palliative Norwood operation [51]; Study of the cardiovascular response in patients with right ventricular bypass and uni-ventricular circulation support [173]; Modelled the cardiovascular control adaptations in chronic renal failure patients [166]; Modelling of the hemodynamic response to hemodialysis induced hypovolemia [54]. Modelling of the cardio-pulmonary response under step-leap respiration exercise for the treatment of patients with Cor Pulmonale [84].

Ventricular assist device support for heart failure	The native cardiovascular system was in heart failure condition, and a VAD model is coupled.	Studies of cardiovascular response in the heart failure condition supported with various types of VADs [70,85,99,174-178]; Studies of cardiovascular response in the heart failure condition supported with intra-aortic balloon pumps [48]; Comparison of the assistance action of different types of VAD and VAD motion profiles [179]; Study of the effect of the inlet and outlet cannulation sites for connecting the VADs to the native cardiovascular system [180]; Study of the physiological control of pulsatility gradient in rotary blood pump [33,181].

Study of cardiovascular response under neuro-regulation	The native cardiovascular system was coupled with the models for the nervous system	Simulate the cardiovascular responses under neuro-regulation in various conditions of isocapnic hypoxia [41,76], hemorrhage [40], hypercapnia and hypocapnic hypoxia [77], carotid occlusion [27]; Simulation of cardiopulmonary response in Valsalva manoeuvre [35]; Simulation of circulation system response to acceleration stress [78]; Simulation of the cardiovascular response to orthostatic stress [22].

Study of special and local circulation loops in the cardiovascular system.	Only the local circulation loop was modelled, and arterial pressure or flow-rate was applied as upstream boundary condition.	Simulation of human foetal cardiovascular system [23]; Studies of cerebral auto-regulation effect [100,103], cerebral vasospasm [80], acute brain damage [102], and cerebral hemodynamics during arterial and CO_2 _pressure change [101]; Modelling of coronary local circulation loop [104]; Study of dependence of intra-myocardial pressure and coronary flow on ventricular loading and contractility [52,93]; Study of venous valves in pressure shielding in the lower extremity [87]; Simulation of venous circulation in lower extremities [37].

As boundary condition in multi-scale simulation of cardiovascular dynamics	The 0D circulation system model was coupled with the distributed parameter models (1D, 2D or 3D).	Multi-scale simulation of the cardiovascular dynamics [147,151,153,159,160].

## 1D cardiovascular models

Propagation of the pressure and flow waves in the vessel network is one of the most intriguing problems in the study of cardiovascular physiology. It is generally believed that valuable information regarding cardiac function, the elastic properties of the vessels, and the patho-physiological conditions of the important organs (brain, liver, kidney etc.) is encoded in these two waveforms and their relationship. Thus pulse wave studies have received extensive attention in cardiovascular research [86].

It can be argued that a model consisting of a series of 0D compartments is, in the limit, a representation of a 1D system, and indeed Milisic and Quarteroni [26] have offered a formal proof that 0D models for the vessel network can be regarded as first order discretisations of one dimensional linear systems. As discussed earlier, such models are readily interpreted in terms of electrical analogues. In practice the biggest difference between multiple linear 0D compartment models and published 1D models is that the latter tend to include the (nonlinear) convective acceleration term whereas the former cannot.

2D and 3D computational fluid dynamics models can reveal the detailed pressure and velocity distribution in a certain segment of the vessel network, but the relatively great demands on computational resource has naturally limited the extent of the domain that is studied. For this situation 1D modelling can offer greater advantages in revealing the pressure and flow changes along the full length of the vessel studied. Canic and Kim [115] have studied in detail the characteristics of the axisymmetric form of the Navier-Stokes equations. They demonstrate that, providing the radius of the vessel is small relative to a characteristic wavelength, the radial momentum equation dictates that the pressure is constant over any cross-section and that, on integrating the axial momentum equation over the cross-sectional area of the vessel, the radial velocity terms are subsumed into an area term. Since this assumption is valid for practical vascular flows, the resulting 1D models, often formulated with flow, pressure and area as the fundamental variables, are considered appropriate for these systems.

Many 1D models for the vessel network have been developed in the past for the study of pulse wave transmission in various applications [116-133]. Generally the model derivation was similar. The differences among these models were mostly in the boundary conditions applied and the solution methods used, and whether and which nonlinear effects were considered for the different applications.

### 1D model derivations

1D pulse wave transmission study in the vessel network is a specific form of fluid-solid interaction. The motion of the blood is governed by the 1D (axisymmetric) form of the incompressible continuity and Navier-Stokes equations, and that of the vessel wall (whether elastic or visco-elastic) is governed by the equations of equilibrium. Most researchers use simple linear or nonlinear constitutive equations to describe the pressure/cross-sectional area relationship [94,116-131,133,134], although more complex models are available [132].

There are several (and some nonlinear) complications in the study of 1D pulse wave propagation, including the tapering of the vessel (causing further variations in convective acceleration), vessel branching, nonlinear pressure/cross-sectional area relationships for the vessel wall, axial tension and bending in the vessel wall, collapse of veins and pulmonary vessels, etc. In deriving the governing equations, most researchers have considered the nonlinear pressure/cross-sectional area relation of the vessel wall by incorporating individually adapted constitutive relations. Brook and Pedly [128], Porental et al. [118], Formaggia et al. [129], Rooz et al. [117], Sherwin et al. [126,134] also included the effect of vessel tapering by considering a varying initial cross-sectional area of the vessel. Vessel collapse was specifically modelled by Brook and Pedley [128], Li and Cheng [121], and Elad et al. [120], by adapting the vessel property and considering the extra-luminal pressure changes for the calculation of collapsed vessel area. Vessel branching as a special boundary condition for segments of vessels is discussed in following sections. Axial tension and bending of the vessel wall have been studied in 2D and 3D cases (e.g. [135]): currently there is no effective implementation in 1D pulse wave studies, although of course axial tethering in a linear system is simply represented by a change in the modulus of the vessel wall.

### Solution methods

The derived governing equations for the 1D pulse wave propagation are a group of hyperbolic equations, and the pulse wave dynamics is determined by the two Riemann invariants [136] (also called the characteristics variables, equal to the blood pressure plus/minus the multiplication of the flow and the characteristic impedance, where the characteristic impedance has the same meaning as the characteristic resistance introduced in previous sections for 0D models) of the system. To solve the governing equations, Steeter et al. [124], Bodley [125], Parker and Joans [116], Wang and Parker [130] and Wang et al. [131] applied the method of characteristics, in which the continuity and momentum equations from the partial differential equations are transformed into ordinary differential equations along the directions of the characteristic lines corresponding to the two Riemann invariants, after which they are readily solved. The governing equations have also been solved using finite difference methods [94,120,121,132,133]. For example, Li and Cheng [121] and Smith et al. [94] discretised the governing equations using the Lax-Wendroff scheme, Elad et al. [120] applied both Lax-Wendroff and MacCormack schemes. In recent years finite volume method and finite element methods have also been applied. Brook and Pedley [128] and Brook et al. [123] used a Godunov scheme to discretise the governing equations in a finite volume formulation. For the finite element formulation, Formaggia et al. [129] adopted the Yoshida projection scheme for the solution of the equations; Wan et al. [127] used a discontinuous Galerkin scheme; Porenta et al. [118] and Rooz et al. [117] applied a Galerkin scheme; and Sherwin et al. [126] solved the equations both with a discontinuous Galerkin scheme and with a Taylor-Galerkin scheme. A small number of researchers have also used spectral method for the solution of pulse wave equations, e.g. Bessems et al. [137] have applied a Galerkin weighted residual method to transform the governing equations into a spectral element space. Sherwin et al. [134] applied the spectral/hp element method.

### Boundary conditions

The governing equations for the 1D pulse wave transmission are hyperbolic in nature, and in the majority of physiological conditions the blood flow is subcritical, and one boundary condition has to be imposed to each end of the vessel [86]. At the upstream side of the vessel, the pressure or flow-rate can be applied based on theoretical derivations or experimental data. For the downstream side, the boundary condition requires more consideration. Most pulse wave transmission studies investigate the blood flow in the arterial network. In physiological conditions, arteries are branched and connected to smaller arterioles, and such branching may continue for many generations. It is impossible to trace all the vessel branching in the simulation and the model must be terminated at some point, depending on the specific aim of the study. Further vessel branches have to be lumped into a suitable terminal description, so that the wave propagation and vessel resistance characteristics of these smaller arterioles can be reasonably represented.

Many researchers have adopted the simplest approach of directly specifying some permissible combination of the pressure and flow-rate at the inlet and outlet boundaries of the vessel [116,120,121,124,125,127,128]. For more realistic downstream conditions, some researchers used constant [117,130] or varying resistance [127], or a three-element Windkessel (RCR model) [118,132,133], to specify the pressure/flow relation at the outlet. Some researchers have chosen to specify the wave reflection coefficient at the outlet as a direct description of a wave propagation feature [119,126,130], or used the non-reflecting boundary condition and compatibility condition [138]. Generally, using a Windkessel as an after-load or directly specifying the wave reflection coefficient have become the most popular downstream conditions in more recent studies. To further improve the accuracy of the downstream boundary condition, Olufsen [122] proposed the structured tree model in which the impedance of smaller arterioles was estimated with linearised Navier-Stokes equations. It was claimed that structured tree model improved the simulation results, but no further validation has yet been published. Smith et al. [94] studied the blood flow in coronary network, and a pressure-dependent vessel network for the coronary arteriole, capillary and venules was used as terminal load to each of the sixth generations of coronary artery branches.

A special kind of boundary condition internal to a vessel segment is that of vessel branching, in which a parent vessel is branched into several daughter vessels. Many researchers directly applied equal static pressures and conservation of flow-rates at the branching points [118,122,127]. An improved description (at least satisfying Bernoulli's equation for the steady state) was adopted by Sherwin et al. [126], who applied continuity of total pressure rather than static pressure. To account for the wave reflection at the branching point, Reymond et al. [133] and Wang and Parker [130] used wave reflection coefficients for the calculation of pressure and flow-rate changes. Smith et al. [94] considered the conservation of momentum at vessel bifurcations by calculating the equilibrium of pressure difference and inertial force.

Another special kind of internal boundary condition for vessel segments is the sudden change of vessel property due to vessel branching, pathological changes, or implantation of prosthetic devices. Rooz et al. [117] and Porenta et al. [118] applied specified pressure drop and continuity of flow-rate in modelling stenotic vessel segment connected with normal vessel. Sherwin et al. [126,134] assumed continuity of flow-rate and total pressure across the interface for the modelling of discontinuous vessel properties. Surovtsova [119] considered the wave reflection at the interface of normal vessels and aortic prosthesis.

### Applications of 1D models

Traditionally the 1D pulse wave transmission models have mostly been applied to study the pulse wave transmission dynamics in arterial segments. The early works of Bodley [125], Streeter et al. [124], Parker and Joans [116] were all of this class. Wang et al. [131], and Wang and Parker [130] later extended the study to investigate the pulse wave dynamics in the various vessel segments in a complete arterial network of the whole human body, including ventricular-arterial coupling. Li and Cheng [121] studied the pulse wave features in the pulmonary arterial network. Porenta et al. [118] and Rooz et al. [117] have studied the pulse wave features in arteries with stenosis. Wan et al. [127] and Steele et al. [139] calculated the pulse wave dynamics in diseased arterial vessels with bypass grafts. Surovtsova [119], Sherwin et al. [126], and Pontrelli and Rossoni [132] studied the pulse wave transmission in stenotic arteries with implanted stents. Reymond et al. [133] modelled the pulse wave propagation in a detailed systemic arterial tree.

Some researchers have studied the pulse wave transmission in collapsible vessels. Elad et al. [120] studied the unsteady fluid flow through collapsible tubes. Brook et al. [128,123] modelled the blood flow in giraffe jugular veins. In such conditions the flow was believed to be supercritical and produced the flow feature of "roll-waves", which was quite different from characteristics of the arterial pulse wave transmission.

Another development in 1D pulse wave transmission modelling is the wave intensity analysis proposed by Park and Joans [116], in which the authors defined the product of pressure and velocity changes over a small interval as the evaluation of rate of energy flux per unit area in a profile of vessel segment. This indicator accurately describes the wave intensity accompanying the pulse wave transmission, and can be used to distinguish the forward transmission wave from the backward transmission wave. The wave intensity analysis has been rigorously applied for the study of pulse wave transmission in the left ventricle [140,141], coronary vessels [142], and systemic arteries [143] and pulmonary arteries [144,145]. However, it is necessary to establish the validity of 1D modelling in such applications before proceeding with the analysis: if the flow is not axially dominated and the secondary flow in the radial and circumferential directions is not negligible, then the credibility of carrying 1D study in such applications will be enormously compromised.

## Multi-scale modelling

The cardiovascular system is a closed network, and there are strong interactions between its components. Emphasis on either only the global circulation dynamics or only the local flow features can provide only partial information of the whole cardiovascular response. In recent years there have been rapid developments in the application of multi-scale modelling techniques, in which 0D models are coupled with 1D, 2D and/or 3D models to form complete representations of the cardiovascular system. Typically now local haemodynamics is computed in an anatomically realistic detailed 3D model of the organ or region of interest, and boundary conditions for this domain are provided by coupled or uncoupled 0D or 1D system models. Quarteroni [146] reviewed the development of cardiovascular system modelling, and gave a basic introduction of the concept of multi-scale modelling. In the multi-scale modelling approaches, the different scales of models have different mathematical characteristics. The 0D lumped parameter models are governed by groups of ordinary differential equations; the 1D distributed parameter models can be described by hyperbolic partial differential equations; the 2D and 3D models are mostly based on the Navier-Stokes equations, which are strongly non-linear partial differential equations whose behaviour may be parabolic, hyperbolic or elliptic, depending on the nature of the specific problems studied. In the solution of multi-scale problems, generally the single scale models are still calculated as they were in the independent studies, whilst at the interfaces special care is required in the handling of the boundary conditions to ensure that the problem is well-posed in the mathematical sense. Because the blood flow in most physiological/pathological conditions is subcritical, one boundary condition needs to be set for each side of every multi-scale model considered. For the 0D model usually the pressure or flow-rate boundary conditions can be directly applied. For the 1D, 2D or 3D distributed parameter models, the boundary conditions can be either prescribed values for variables in the governing equations, or prescribed values for the derivatives of variables, or prescribed values for the linear combination of variables and their derivatives. The 0D and 1D models use pressure and velocity (or flow rate) as basic variables, spatially averaged (or integrated) over the transverse plane (as discussed earlier, uniform pressure on a cross-section is a consequence of the radial momentum equation, whilst the velocity distribution in the nonlinear convective term can be represented by a correction coefficient [115]). The 2D and 3D models often utilise pressure and velocity as primitive variables.

A multi-scale model description will include a strategy for the coupling of the single scale components, and many such strategies are possible. Pontrelli [147] coupled a 1D arterial pulse wave transmission model to two 0D compartment models representing the components upstream and downstream of the 1D section. The upstream 0D model provided the inlet flow-rate and the downstream 0D model the outlet pressure as boundary conditions for the 1D model, whilst the 1D model returned upstream pressure and downstream flow-rate as boundary conditions for the appropriate 0D models. In a similar study, Formaggia et al. [148] embedded a 1D descending aorta model in a 0D systemic loop. In the study the converse of the above coupling strategy was applied. An additional issue that arises when coupling to 2D or 3D models is that of dealing with the problem of defective boundary conditions: the lack of distribution information makes the flow-rate description in 0D/1D models insufficient to be applied as boundary conditions for 2D/3D models. Possible solutions include mapping of flow-rates from 0D/1D models to a velocity distribution based on Womersley's solutions [149,150] for transient flows in a long tube. Watanabe et al. [151] carried out multi-scale simulation of left ventricular filling dynamics, in which a 3D ventricular blood flow model was integrated with a 0D model for the other parts of the circulation system. The 0D model provided the pressure as boundary conditions for the 3D model, and the 3D model specified the flow rate changes for the 0D model. Vigono-Clementel et al. [152] applied 0D vessel network as terminal loads to a complex 3D arterial branching model. The 0D model provided the resistance/impedance value as boundary conditions to the 3D arterial model by supplying the pressure-flow rate relation at the model interfaces. At the model interfaces, the outlet velocity profile for the 3D model was represented as Poiseuille flow for the resistance boundary condition, and as Womersley's linear wave solution for the impedance boundary condition. In assessing two different operation techniques on the postoperative haemodynamics in the treatment of hypoplastic left heart syndrome, Migliavacca et al. [153] embedded a 3D model of a systemic to pulmonary conduit in a 0D multiple branched circulation system model. In the upstream interface for the 3D model, total pressure (static pressure plus the kinetic head) calculated from the 0D model was applied as the boundary condition, and the inlet velocity was assumed to be normal to the boundary, whereas at the downstream interface the static pressure from the 0D model was specified as the boundary condition. In an analysis of blood flow in compliant vessels, Formaggia et al. [129] coupled a 1D model to the 3D model to reduce the computational complexity and to remove the effect of the outgoing pressure waves. To derive the missing information of variable distributions at the model interface, they proposed the usage of two approaches to find the admissible pressure or velocity distribution: a variational approach and a Lagrange multiplier approach. In other studies, Formaggia et al. [25,154] further elaborated the two approaches and suggested that for transient flow problems, the Womersley profile is an admissible flow distribution. Vigono-Clementel et al. [152] adopted this treatment of the model interfaces.

## Conclusions and Recommendations

This review examines published 0D and 1D time domain cardiovascular models, and provides an overview of their development and applications, as well as their emerging role in multi-scale modelling. Configurations of the 0D models are becoming ever more sophisticated and advanced, and the various developed models have seen wide and successful applications in the study of cardiovascular physiology, evaluation of new artificial cardiovascular devices, aeronautic medicine and more. 1D models were mostly confined to the study of arterial haemodynamics, where their ability to capture wave transmission effects is important, with some minor extensions to venous dynamics. 1D models have been successfully applied in the context of clinical diagnosis of pathological changes in the cardiovascular system (such as hypertension, atherosclerosis), and in the context of stent design.

Models are developed to achieve specific research purposes in each individual studies, thus the complexity of the models should fit the purposes of the studies. An over-simplified model will produce inadequate accuracy in the study. However, this does not mean that more complex model will always produce more accurate results. For example, if the purpose of the study is to evaluate the short term assist action of a ventricular assist device on the failing heart, and the neuro-regulation effect does not need to be considered, then a single branch multiple-compartment model for the systemic vasculature, in which the vascular is divided into the aorta, artery, arteriole, capillary and vein segments, is sufficient to work as the after-load to the assisted heart. It is not necessary to model every artery and venous branches in this situation, since in an overly detailed vessel branch model the parameter setting becomes quite difficult. However, if the purpose of the study is to simulate the acceleration stress in flight training, then it is quite necessary to model the vessel branch for the lower extremity separately in order to include the effect of blood pooling, besides specifically coupling the neuro-regulation action in the cardiovascular model. There is no universally optimal model that suits every application. Researchers must decide what level of model sophistication is most suitable in their specific studies.

Since 0D model is a high level abstraction of the circulatory system and one model component often represents several complex anatomical structures, proper setting of model parameters is an important issue to be seriously addressed. As discussed in previous sections, this is still an area not sufficiently explored, with inconsistent model parameters being adopted among different researchers. Further effort in this direction is very necessary before a cardiovascular model can be considered as fully validated.

Previous studies have suggested a number of cardiovascular effects that need to be specially addressed in the 0D cardiovascular modelling, including ventricular interaction, effect of pericardial, atrial-ventricular interaction, auto-regulation in some local circulation loops, auxiliary pumping action to blood flow caused by peripheral muscle contraction, venous valve in some vessels such as in the lower extremity etc. Although intuitively correct, until now these effects have undergone very limited validations, due to the difficulty in isolating each of these effects from the overall cardiovascular response (which is often a coupled interaction among the different organs) as well as the restrictions in *in vivo *measurements. In the future effort should be made to further quantify the relative importance of each of these effects in the overall cardiovascular response, and to further study their underlying mechanism as well as to find proper parameter settings in their modelling.

The circulatory system does not work in isolation. It has close interaction with other systems such as the nervous system, respiratory system, and digestive system. Study of their coupled reactions, such as cardiovascular response under neuro-regulation and hormone control, coupled cardio-pulmonary response, simulation of coupled circulatory dynamics and transportation of nutrients/metabolic remaining, will bring the 0D cardiovascular modelling to a higher level, and such results will enormously improve our quantitative understanding of human physiology. Examples of models that address these complex interactions are those by [40,84]. Further effort should be continued and this is a field with very promising prospects.

0D cardiovascular models, especially the more complex ones, were often developed for research purposes. So far only concepts of vascular impedance and pulse wave velocity are widely used to assist clinical diagnosis and treatment, and few integrated 0D model comprising the complete description of heart and vessels have seen use in clinical practice. With the success of 0D models in simulating cardiovascular dynamics under various physiological and pathological conditions, as discussed in previous sections, it is time to work with the clinical community to personalise the 0D integrated models to achieve patient-specific modelling, and this will bring innovations to the cardiovascular clinical practice. To minimise the difficulty in parameter setting, models for patient-specific analysis may have reduced complexity as compared to those for research purposes.

The majority of current 1D cardiovascular models assume a uniform vessel property. Although the non-linear effects, such as the vessel tapering and curvature, visco-elastic property and the inertia of the vessel etc., have been analysed previously [125], these were not universally considered in the 1D models. With sufficient experience gained during the past decades on the 1D modelling using uniform vessel property, it is time to seriously consider these non-linear effects in the future 1D modelling.

The majority of researchers neglected the inertial of the vessel wall in the 1D cardiovascular modelling. There are a small number of researchers [155-158] who specifically included this factor in their 1D model derivation, and thus arrived at a wave dynamics equation in the form of the Korteweg-de Vries equation, and the corresponding wave dynamics is governed by a special kind of wave called solitary wave. It was claimed that the solitary wave is a better description of the arterial pulse wave since it matches the experimental results better than the hyperbolic wave equation that is currently used by the majority of researchers. This seems to be an interesting area that deserves more exploration.

Some researchers have developed sophisticated 1D models for the arterial tree [133,159], covering those major arterial branches in the vessel anatomy. By assigning realistic diameter, length, thickness and elasticity values to the individual vessel segments and blood density values, physiologically realistic pulse wave transmission characteristics have been successfully simulated in the model. Such models and results will be useful to assist the parameter setting in 0D models. Future effort may be made in this area for the development of valid techniques to improve the parameter settings in 0D modelling.

With the development of computer hardware and numerical analysis techniques, higher dimensional haemodynamic analysis (2D and 3D) using computational fluid dynamics is no longer a prohibitive task. Thus to address the requirement of high accuracy and ability to simulate the interaction among cardiovascular organs concurrently, it is possible and necessary to couple the 0D models and the 1D/2D/3D models to build multi-dimensional models. Some successful attempts have been made in this direction [129,160,161]. Also some modelling methodologies in this area have been patented or are under patent application [162,163]. However, treatment of domain boundaries among the different dimensional models within an overall framework still needs further improvement. This includes not only the matching of mean pressure/flow values and their distribution information on the domain interfaces, but also the correct wave reflection description. Breakthroughs to be made on this issue will be enormously helpful to the improvement of simulation accuracy. This issue is one of the research topics in the EU funded euHeart project [164].

**Figure 6:**
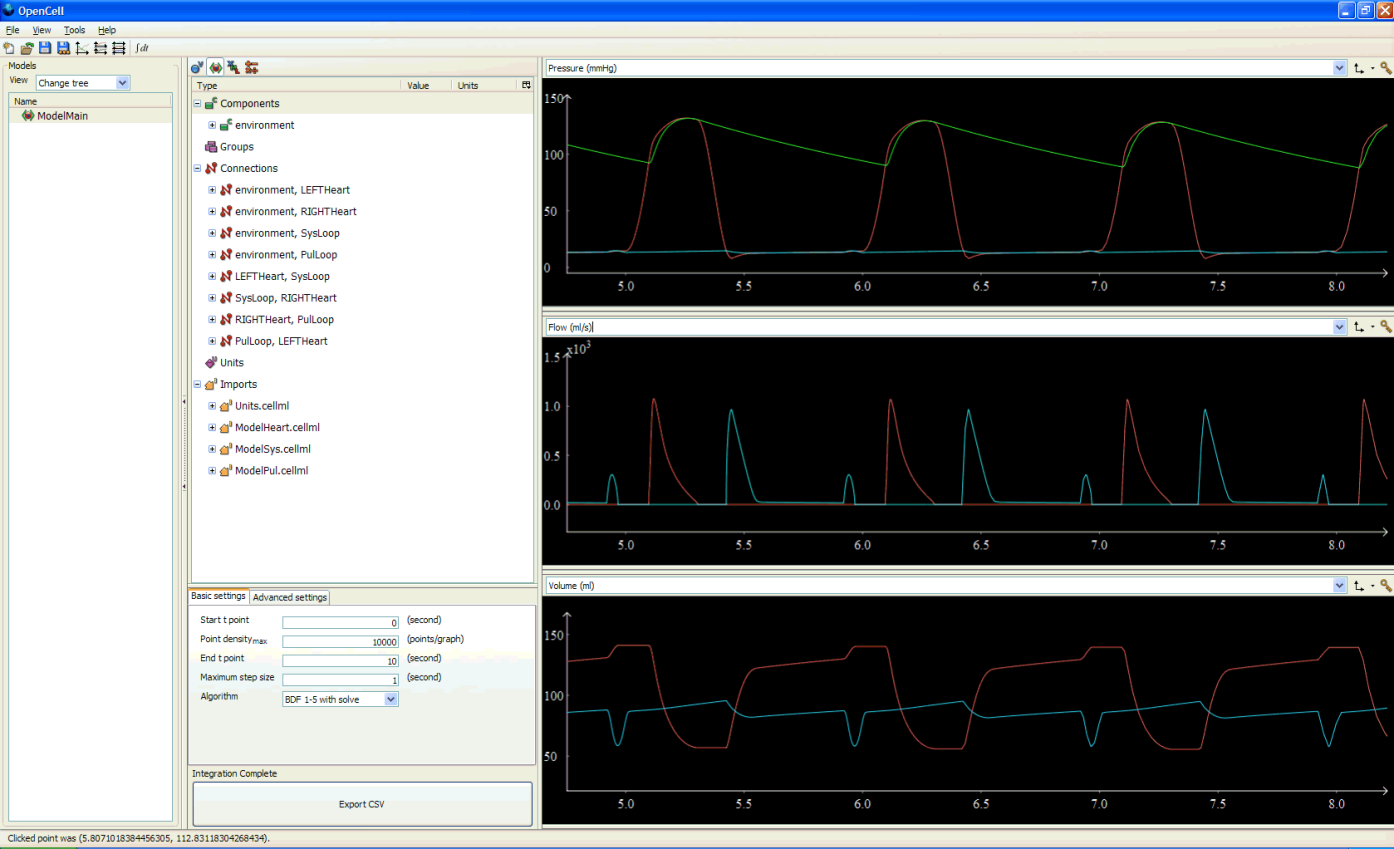
**Screen capture of a 0D cardiovascular model running in the OpenCell environment, simulating the circulatory response in a healthy human subject**.

As a conclusion of the paper, the authors' group has developed a series of 0D cardiovascular models with different complexities, which can be used for education purposes as well as used as a foundation for developing more advanced models. These models have been represented in the CellML mark-up language, and are freely available for download by the research community from the CellML model repository [112]. Using an accompanying tool called OpenCell in the CellML website, the models can also be export into subroutines in C, Python, Matlab and some other mainstream programming languages, and used in undergraduate teaching. Figure 6 shows the screen snapshot of one of these CellML models running in the OpenCell environment, simulating the circulatory response in a healthy human subject. The right panel of the figure illustrates the pressure, flow and volume changes simulated.

## Competing interests

The authors declare that they have no competing interests.

## Authors' contributions

YS drafted the paper. RH revised the paper and gave final approval of the version to be published. PL revised the paper. All authors read and approved the final manuscript.

## Glossary

Acceleration stress: Physiological changes that occur in the human body in motion as a result of rapid increase of speed. Rapid acceleration and surges in acceleration are felt more critically than are gradual shifts. Pilots are especially subject to the effects of acceleration because of the high speeds at which they travel. Acceleration forces are measured in units of gravitational acceleration, or g. A force of 3 g, for example, is equivalent to an acceleration three times that of a body falling near Earth.

After-load: The pressure, or resistance, against which the left ventricle must eject its volume of blood during contraction. The resistance is produced by the volume of blood already in the vascular system and by the constriction of the vessel walls. Whether the after-load means the pressure or the resistance depends on the context it is used.

Elastance: An expression of the measure of the ability to do so in terms of unit of volume change per unit of pressure change.

Frank-Starling effect: The effect that the heart changes its force of contraction and therefore stroke volume in response to changes in venous return. It is also called the Frank-Starling mechanism.

Guyton's model: a very comprehensive cardiovascular model which include not only the haemodynamic description for vessel branches, but also the regulation model for the autonomous and the hormone systems. (Guyton, A. C., Coleman, T. G. & Granger, H. J. 1972 Circulation: overall regulation. Annu Rev Physiol 34, 13-46.)

Laplace law: A principle of physics that the tension on the wall of a sphere or cylinder is the product of the pressure times the radius of the chamber and the tension is inversely related to the thickness of the wall.

Multi-scale model: a model that includes different dimensions of descriptions for different parts of the model. For sample models please refer to (Formaggia, L., Nobile, F., Quarteroni, A. & Veneziani, A. 1999 Multiscale modelling of the circulatory system: a preliminary analysis. Computing and Visualization in Science 2, 75-83.).

Orthostatic stress: Means the increased stress developed in the circulatory system during upright standing while relieved by recumbency. This is often observed as variations in blood flow and blood pressure regulation. Standing successfully requires interplay of blood volume, physical, neurologic, hormonal, and vascular factors which compensate for the effects of gravity on venous pooling. Patients with orthostatic intolerance have compensatory dysfunction in these factors and thus demonstrate symptoms of lightheadedness, headache, fatigue, neurocognitive disorders, visual disturbances (black/white/spots), hyperpnea/dyspnea, tremulousness, sweating, anxiety/palpitations during upright standing.

Pulmonary loop: the vessels that formed the pulmonary circulation, including pulmonary arteries, pulmonary arterioles, pulmonary capillaries, pulmonary venules, and pulmonary veins.

Stroke volume: The volume of blood pumped from one ventricle of the heart with each beat.

Stroke work: the work done by the ventricle to eject a volume of blood (i.e., stroke volume) into the aorta.

Systemic circulation: the part of the cardiovascular system which carries oxygenated blood away from the heart to the body, and returns deoxygenated blood back to the heart.

Systemic loop: the vessels that formed the systemic circulation, including aorta, systemic arteries, systemic arterioles, systemic capillaries, venules, veins, and vena cava.

Subcritical flow: A flow condition in which the flow velocity is smaller than the wave velocity.

Supercritical flow: A flow condition in which the flow velocity is larger than the wave velocity.

Vascular impedance: Defined as the ratio of pressure to flow at a specific position of the blood vessel, which is a measure of the impediment offered to blood flow at the defined position by the downstream vasculature. Vascular impedance is a combined effect produced by the friction to blood flow, vessel elasticity, and the blood inertia in the downstream vasculature.
